# Incidence and trends for notifiable infectious diseases in Shenyang, China, 2005–2024

**DOI:** 10.1186/s12889-026-27619-3

**Published:** 2026-05-07

**Authors:** Huijie Chen, Huiyu Wen, Ye Chen, Lihai Wen, Zhuo Jin, Bingzheng Zhou

**Affiliations:** 1 Department of Infectious Disease, Shenyang Municipal Center for Disease Control and Prevention (Shenyang Municipal Health Supervision Institute), Shenyang, Liaoning Province PR China; 2 Department of Epidemiology, Shenyang Medical College, Shenyang, Liaoning Province PR China; 3 Center of Reproductive Medicine, Department of Obstetrics and Gynecology, Shengjing Hospital of China Medical University, Shenyang, Liaoning Province PR China; 4 Department of Orthopedics and Sports Medicine, Shengjing Hospital of China Medical University, Shenyang, Liaoning Province PR China

**Keywords:** Notifiable infectious diseases, Incidence, Trends, Shenyang

## Abstract

**Background:**

Infectious diseases remain a major global public health challenge. Evolving epidemiological patterns and considerable regional variation underscore the need for localized, real-time surveillance and assessment. This study examines the incidence and trends of notifiable infectious diseases (NIDs) in Shenyang from 2005 to 2024 to generate an evidence base for regional and national public health strategy formulation.

**Methods:**

The data of NIDs in Shenyang from January 1, 2005 to December 31, 2024 were obtained from the National Notifiable Diseases Reporting System (NNDRS). Joinpoint regression analysis was used to determine the changing trend of annual incidence.

**Results:**

Between 2005 and 2024, Shenyang reported 668,476 cases of 33 notifiable infectious diseases, all classified as Class B (58.8%) or C (41.2%). Respiratory infections were the most common (47.4%), followed by fecal-oral (29.8%) and blood-borne/sexual transmission (21.7%). The overall annual age-standardized incidence rate (ASIR) averaged 481.49 per 100,000 population, increasing significantly from 360.72 to 880.18 per 100,000 over the 20-year period (AAPC: 6.20%; 95%CI: 2.11%,10.04%), representing a 144% overall rise. Males exhibited a higher average annual ASIR (565.30 per 100,000) compared to females (393.35 per 100,000). A pronounced upward trend was observed in Class C diseases (AAPC: 12.75%; 95%CI: 7.47%,18.19%), whereas Class B diseases remained stable. Respiratory-transmitted diseases showed the strongest growth (AAPC: 12.75%; 95%CI: 7.47%,18.19%), followed by those transmitted via the fecal-oral route (AAPC: 4.79%; 95%CI: 0.42%,9.65%), while blood-borne/sexually transmitted diseases declined slightly (AAPC: -2.52%; 95%CI: -4.11%,-0.30%). Vaccine-preventable diseases (VPDs) declined markedly (AAPC: -6.98%; 95%CI: -8.62%,-5.30%), in contrast to a substantial increase in non-VPDs (AAPC: 13.66%; 95%CI: 10.04%,17.68%).

**Conclusions:**

The overall incidence of NIDs in Shenyang was lower than the national average, however, the respiratory infections pose a relatively high disease burden and warrant focused control. It is noteworthy that the incidence of VPDs has declined significantly over the past two decades, suggesting a possible beneficial impact of immunization strategies.

## Background

Infectious diseases continue to pose a major global public health threat, contributing to substantial morbidity, mortality, and economic losses each year [1, 2]. Despite progress in medical technology and healthcare infrastructure, the recurrent emergence and re-emergence of pathogens such as SARS-CoV-2 underscore the ongoing vulnerability of human populations to infectious disease outbreaks [3, 4]. According to the World Health Organization (WHO), millions of annual deaths are attributable to infectious diseases, with low- and middle-income countries carrying a disproportionately high burden [5]. In China, such diseases have long constituted a significant part of the national disease profile, leading to the implementation of robust surveillance mechanisms including the National Notifiable Diseases Reporting System (NNDRS) in 2004 [6].

Globally, the epidemiology of infectious diseases has undergone considerable changes over the past two decades [7]. The incidence of vaccine-preventable diseases (VPDs) including measles, poliomyelitis, and pulmonary tuberculosis (PTB) has declined in many parts of the world [8–12], and the global burden of enteric infections also exhibited a downward trend between 1990 and 2021 [13]. In contrast, sexually transmitted infections have increased markedly, influenced by a combination of biological and socio-behavioral factors, with marginalized and high-risk groups-especially in resource-limited settings-being disproportionately affected [14–16]. Furthermore, growing international travel and migration have contributed to a modest resurgence of malaria in certain areas [17]. The COVID-19 pandemic disrupted longstanding seasonal influenza dynamics. After the relaxation of non-pharmaceutical interventions (NPIs) such as international travel restrictions, social distancing, mask mandates, and school closures, some countries witnessed a rapid rebound in influenza activity, whereas others reported delayed or off-season epidemics [18, 19].

In China, the overall incidence of notifiable infectious diseases (NIDs) fell markedly between 1975 and 1995, largely attributable to expanded immunization coverage and improved public sanitation [20]. However, a gradual resurgence occurred from 1995 to 2008, fueled by increases in blood-borne/sexually transmitted diseases-such as hepatitis B and syphilis, as well as respiratory infections including PTB and influenza [20]. Recent studies covering data from 2005 to 2024 indicated an average annual incidence of 549.76 per 100,000 population, with an overall upward trajectory [21, 22].

Regionally, China’s vast territory contributes to distinct epidemiological characteristics of NIDs. For example, in Nanjing, Jiangsu Province, the average annual incidence of NIDs from 2004 to 2022 was 347.45 per 100,000 population, with fecal-oral transmission being the dominant route [23]. No significant temporal trend in annual incidence was observed over the past two decades [23]. However, during the same period, Henan Province recorded a significantly higher average annual incidence of 417.47 per 100,000, predominantly driven by blood-borne/sexual transmission [24]. Furthermore, the annual incidence in Henan exhibited a clear upward trend over the last 20 years [24].

Economic development has accelerated population mobility, amplifying the risks of infectious disease transmission, particularly for respiratory and blood-borne pathogens [25, 26]. The dynamic nature of epidemiological trends necessitates continuous reinforcement of surveillance systems. China’s substantial regional heterogeneity in disease epidemiology underscores the critical importance of localized research. Shenyang, a major city in Liaoning Province with a population exceeding eight million, acts as a vital surveillance sentinel for monitoring regional infectious disease patterns. However, a comprehensive analysis of long-term trends in this context remains lacking [27], limiting the development of precisely targeted prevention strategies. This study aims to address this gap by examining the 20-year epidemiological trends of NIDs in Shenyang, with a focus on identifying local distinctive patterns (e.g., shifts in dominant transmission routes and disease spectrum) that may diverge from national averages, thereby providing an evidence base to inform regional public health strategies.

## Methods

### Study area

Shenyang, the capital of Liaoning Province in China, is situated between latitudes 41°11′N and 43°02′N, and longitudes 122°25′E and 123°48′E. As a prefecture-level city, it covers an area of 12,860 square kilometers and comprises 13 districts. From 2005 to 2024, the average population of Shenyang was 8,151,603.

### Data sources

In China, the list of NIDs has undergone dynamic adjustments to address emerging public health threats. Foot and mouth disease (HFMD) was first incorporated in 2008, followed by influenza A(H1N1) in 2009, which was later integrated into seasonal influenza surveillance in 2014. Subsequently, H7N9 avian influenza was added in 2013 and reclassified as human infection with novel influenza subtype in 2018 to encompass other novel subtypes. More recently, COVID-19 and monkeypox (Mpox) were included as statutory notifiable diseases in 2020 and 2023, respectively. Currently, there are totaling 40 NIDs in China, which were classified into classes A, B and C [28] (Table 1).

**Table 1:** Current NIDs and classification in China

Classification	No. of diseases	NIDs
A	2	Plague and cholera
B	27	COVID-19, AFP, rabies, anthrax, meningococcal meningitis, scarlet fever, leptospirosis, SARS, human infection with a novel influenza A virus subtype, JE, bacillary and amebic dysentery^§^, HB, schistosomiasis, HIV/AIDS, measles, dengue fever, PTB, diphtheria, gonorrhea, malaria, viral hepatitis^∮^, HFRS, Mpox, typhoid and paratyphoid fevers^$^, neonatal tetanus, syphilis
C	11	Influenza, leprosy, filariasis, mumps, epidemic and endemic typhus, hand, HFMD, rubella, visceral leishmaniasis, other infectious diarrhea^£^, AHC, echinococcosis

*AFP* Acute flaccid paralysis, *AHC* Acute hemorrhagic conjunctivitis, *HB* Human brucellosis, *HFMD* Hand, foot and mouth disease, *HFRS* Hemorrhagic fever with renal syndrome, *JE* Japanese encephalitis, *Mpox* Monkeypox, *NIDs* Notifiable infectious diseases, *PTB* Pulmonary tuberculosis, *SARS* Severe acute respiratory syndrome

^§^Bacillary and amebic dysentery comprises two distinct diseases: bacillary dysentery and amebic dysentery. ^∮^Viral hepatitis comprises six distinct disease types: Hepatitis A, Hepatitis B, Hepatitis C, Hepatitis D, Hepatitis E, and hepatitis (untyped). ^$^Typhoid and paratyphoid fevers comprise two distinct diseases: typhoid fever and paratyphoid fever. ^£^Other infectious diarrhea refers to diarrhea other than that caused by cholera, dysentery or typhoid fever

The NNDRS was established and officially put into operation in April 2004.This study collected all reported cases (including time of onset, diagnosis, reporting date, as well as patient demographic details such as age and gender) with onset from January 1, 2005 to December 31, 2024, in Shenyang, China through this system which was used under license and not publicly available. All eligible cases were confirmed based on clinical manifestations and laboratory tests, and the following cases were excluded: not residing in Shenyang, duplicate reported and without a definitive diagnosis.

The annual population data for the years 2005–2024 were retrieved from the Official Website of Shenyang Statistical Bureau (http://tjj.shenyang.gov.cn/). The study protocol was approved by the Ethics Committees of Shenyang Center for Disease Control and Prevention.

### Definition

Respiratory transmitted infectious diseases are defined as a category of illnesses acquired through exposure to respiratory droplets generated by infected individuals during coughing or sneezing, or via contact with contaminated surfaces (fomites) [29]. This category in the present study includes COVID-19, severe acute respiratory syndrome (SARS), influenza, meningococcal meningitis, scarlet fever, human infection with a novel influenza A virus subtype, pertussis, measles, PTB, leprosy, mumps, rubella, and diphtheria.

Infectious diseases transmitted via the fecal-oral route are defined as those caused by pathogens that are shed in the feces of infected individuals and subsequently enter a new susceptible host through the mouth via intermediaries such as contaminated water, food, or hands [30]. This transmission group in our study covers acute flaccid paralysis (AFP), bacillary and amebic dysentery, typhoid and paratyphoid fevers, hepatitis A, hepatitis E, HFMD, and other infectious diarrhea.

Blood-borne/sexually transmitted infectious diseases refer to a category of illnesses contracted through exposure to infected blood or body fluids during sexual contact, sharing of injection equipment, or other forms of direct exposure [31]. Diseases classified under this mode in the current analysis include HIV/AIDS, gonorrhea, syphilis, hepatitis B, hepatitis C, hepatitis D, and hepatitis (untyped).

Zoonotic and vector-borne infectious diseases are caused by pathogens naturally transmitted between vertebrate animals and humans, either directly (zoonoses) or indirectly via arthropod vectors such as mosquitoes and ticks [32, 33]. In this study, these include rabies, cutaneous anthrax, human brucellosis (HB), hemorrhagic fever with renal syndrome (HFRS), echinococcosis, Japanese encephalitis (JE), dengue fever, malaria, typhus, filariasis, and visceral leishmaniasis.

VPDs are those targeted by national immunization programs for control through vaccination [8]. The VPDs considered here comprise measles, pertussis, meningococcal meningitis, JE, AFP, hepatitis A, hepatitis B, PTB, rubella, and mumps. All remaining diseases are categorized as non-VPDs.

### Statistical analysis

Disease incidence was measured using the age-standardized incidence rate (ASIR). The annual ASIR was computed as the sum of the products of age-specific incidence rates and the corresponding standard population weights for each age group in a certain year. In this study, the annual ASIR from 2005 to 2024 were calculated and the 2010 national population census of China was serving as the standard population. The changes in annual ASIR from 2005 to 2024 were calculated as the percentage change, using the formula: $\:\frac{{ASIR}_{2024}-{ASIR}_{2005}}{{ASIR}_{2005}}\times\:100\%$. The average annual ASIR was defined as the mean of the annual ASIR for each year during the observation period. The average annual percent change (AAPC) and its corresponding 95% confidence interval (CI) were calculated. The AAPC quantifies the average direction and magnitude of a trend over the entire study period. If the 95%CI for the AAPC did not include zero, the trend was considered statistically significant. In such cases, the trend was classified as “increasing” for a positive value or “decreasing” for a negative value. Conversely, if the 95%CI contained zero, the trend was deemed “stationary”, indicating that the null hypothesis (AAPC = 0) could not be rejected. A two-sided *P* < 0.05 was considered statistically significant.

Stata version 17.0 (Stata Corp, College Station, TX, USA) was used for data extraction, sorting, and cleaning. Microsoft Excel 2019 (Microsoft, Seattle WA, USA) was utilized to visualize the epidemic patterns. The Joinpoint Regression Program, version 4.8.0.1 (National Cancer Institute, Bethesda, MD, USA) was performed to analyze temporal trends.

## Results

### General characteristics

A total of 1,018,131 cases of infectious diseases were reported from 2005 to 2024. Following the application of exclusion criteria, 184,033 cases were excluded due to non-residency in Shenyang. An additional 1,733 cases were identified as duplicate reports and were subsequently removed. Furthermore, 57,483 cases were diagnosed as other conditions, and 106,406 cases were categorized as non-NIDs. The latter category comprised 89,384 cases of varicella, 6,513 cases of condyloma acuminatum, 4,358 cases of genital chlamydia trachomatis infection, 3,453 cases of tuberculous pleurisy, 53 cases of clonorchiasis, 1,607 cases of genital herpes, 1,015 cases of non-gonococcal urethritis, 17 cases of severe fever with thrombocytopenia syndrome, 4 cases of extrapulmonary tuberculosis, 1 case of streptococcus suis infection, and 1 case of scrub typhus. Consequently, the final analytical dataset comprised a total of 668,476 cases.

### Proportion and dynamics

**Fig. 1:**
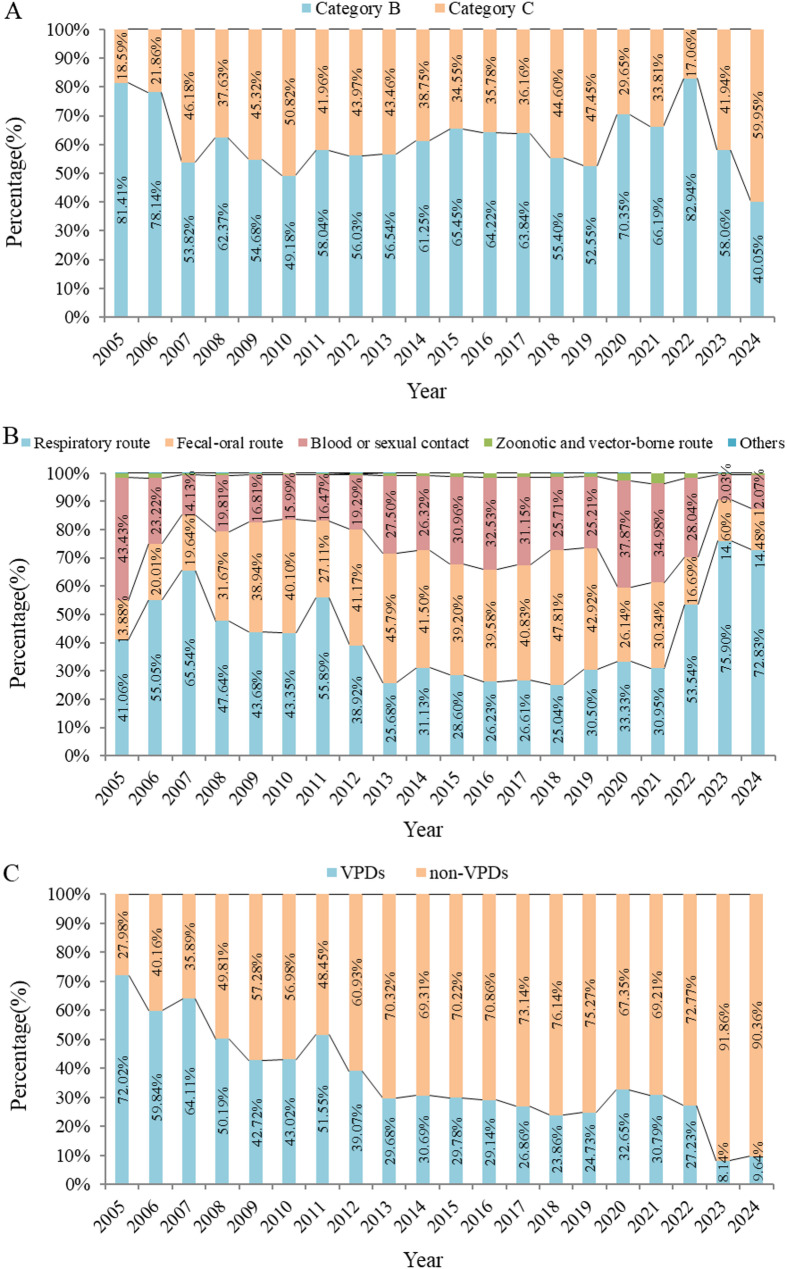
Proportion of NIDs in Shenyang, China by different categories from 2005–2024. **A** Percentage of category B and category C. **B** Percentage of five transmission routes. **C** Percentage of VPDs and non-VPDs. The colors of the bars denote different categories, while their length indicates each category’s percentage share of the total

As summarized in Tables 2 and 33 NIDs were reported in Shenyang from 2005 to 2024, consisting of 24 Category B and 9 Category C diseases. No Category A infections were documented, and no cases of leptospirosis, SARS, diphtheria, filariasis, or visceral leishmaniasis were recorded. Of all the reported NIDs, Category B diseases accounted for 393,215 cases (58.82%) and Category C diseases accounted for 275,261 cases (41.18%). As shown in Fig. 1, with the exception of 2010 and 2024, Category B diseases consistently represented a higher proportion of annual cases than Category C diseases, exceeding 80% of the total in both 2005 and 2022.

**Table 2:** Proportion of NIDs reported in Shenyang, 2005–2024

	Respiratory route	No.of cases(%)	Fecal-oral route	No.of cases(%)	Blood-borne/sexual route	No.of cases(%)	Zoonotic and vector-borne route	No.of cases(%)	Other route	No.of cases(%)	Total(%)
Category B	PTB	100,866(15.09%)	Bacillary and amebic dysentery	38,848(5.81%)	Syphilis	62,635(9.37%)	HB	5299(0.79%)	Mpox	10(0.00%)	393,215(58.82%)
	COVID-19	58,633(8.77%)	Hepatitis E	6326(0.95%)	Hepatitis B	43,951(6.57%)	HFRS	1580(0.24%)	Neonatal tetanus	1(0.00%)	
	Scarlet fever	28,806(4.31%)	Hepatitis A	2929(0.44%)	Hepatitis C	12,701(1.90%)	Malaria	97(0.01%)	Schistosomiasis	1(0.00%)	
	Measles	4262(0.64%)	Typhoid and paratyphoid fever	115(0.02%)	Hepatitis D	5(0.00%)	Anthrax	75(0.01%)	-	-	
	Meningococcal meningitis	78(0.01%)	AFP	82(0.01%)	Gonorrhea	10,255(1.53%)	Dengue fever	44(0.01%)			
	Pertussis	60(0.01%)	-	-	HIV/AIDS	9885(1.48%)	JE	5(0.00%)			
	Human infection with a novel influenza A virus subtype	2(0.00%)			Hepatitis (untyped)	5662(0.85%)	Rabies	2(0.00%)			
Subtotal		192,707(28.83%)		48,300(7.23%)		145,094(21.71%)		7102(1.06%)		12(0.00%)	
Category C	Influenza	66,475(9.94%)	HFMD	93,466(13.98%)	-	-	Epidemic and endemic typhus	79(0.01%)	AHC	42(0.01%)	275,261(41.18%)
	Mumps	35,639(5.33%)	Other infectious diarrhea	57,464(8.60%)			Echinococcosis	5(0.00%)	-	-	
	Rubella	22,090(3.30%)	-	-			-	-			
	Leprosy	1(0.00%)									
Subtotal		124,205(18.58%)		150,930(22.58%)				84(0.01%)		42(0.01%)	
Total		316,912(47.41%)		199,230(29.80%)		145,094(21.71%)		7186(1.07%)		54(0.01%)	668,476(100%)

*AFP* Acute flaccid paralysis, *AHC* Acute hemorrhagic conjunctivitis, *HB* Human brucellosis, *HFMD* Hand, foot and mouth disease, *HFRS* Hemorrhagic fever with renal syndrome, *JE* Japanese encephalitis, *Mpox* Monkeypox, *NIDs* Notifiable infectious diseases, *PTB* Pulmonary tuberculosis

In this table, the subtypes of viral hepatitis are presented separately to allow for comparison based on their distinct transmission routes. Additionally, influenza A (H1N1) was grouped with seasonal influenza for analysis

In terms of transmission routes, respiratory transmission was the most common (316,912 cases, 47.41%), followed by fecal-oral (199,230, 29.80%) and blood-borne/sexual transmission (145,094 cases, 21.71%). Zoonotic and vector-borne transmissions accounted for 7,186 cases (1.07%), with other routes comprising 54 cases (0.01%). As shown in Fig. 1, the dominant transmission routes shifted considerably over this period. Blood-borne/sexual transmission predominated in 2005, followed by respiratory transmission during 2006–2011, and fecal-oral transmission during 2012–2019. Since 2020, respiratory transmission has reemerged as the primary route, comprising over 70% of cases in both 2023 and 2024.

VPDs accounted for 209,962 cases (31.41%) during the 20-year period, while non-VPDs comprised 458,514 cases (68.59%). As shown in Fig. 1, a pronounced decline in the proportion of VPDs was observed over time, falling from 72.02% in 2005 to just 8.14% in 2023 and 9.64% in 2024.

PTB was the most reported disease (100,866 cases, 15.09%), followed by HFMD (94,466 cases, 13.98%), viral hepatitis (71,574 cases, 10.71%), and influenza (66,475 cases, 9.94%). Substantial case numbers were also noted for syphilis (62,635 cases, 9.37%), COVID-19 (58,633 cases, 8.77%), and other infectious diarrhea (57,464 cases, 8.60%). In contrast, neonatal tetanus, schistosomiasis, and leprosy were the least reported, with one case each. Other rarely reported diseases included hepatitis D, JE, and echinococcosis (5 cases each), human infection with a novel influenza A virus subtype and rabies (2 cases each), as well as typhoid and paratyphoid fever (115 cases), malaria (97 cases), AFP (82 cases), epidemic and endemic typhus (79 cases), meningococcal meningitis (78 cases), anthrax (75 cases), pertussis (60 cases), dengue fever (44 cases), AHC (42 cases), and Mpox (10 cases).

### Incidence and temporal trends

Table 3 summarizes the incidence and trends of NIDs in Shenyang from 2005 to 2024. The overall average annual ASIR was 481.49 per 100,000 population, showing a significant upward trend with an AAPC of 6.20% (95% CI: 2.11%,10.04%). The ASIR increased substantially from 360.72 per 100,000 in 2005 to 880.18 per 100,000 in 2024, corresponding to an overall rise of 144.01% over the 20-year period.

**Table 3:** Incidence and trends of NIDs in Shenyang, 2005–2024

	ASIR(/100,000)		Change of ASIR(%)	AAPC(%,95%CI)	Average annual ASIR(/100,000)
	2005	2024			
All	360.72	880.18	144.01	6.20^*^(2.11,10.04)	481.49
Male	444.96	937.66	110.73	5.27^*^(1.28,8.95)	565.3
Female	268.92	839.93	212.33	7.69^*^(3.36,11.93)	393.35
Category B	275	292.58	6.39	2.41(-1.45,5)	243.4
Category C	85.72	587.6	585.49	12.75^*^(7.47,18.19)	238.09
VPDs	259.68	68.23	-73.73	-6.98^*^(-8.62,-5.3)	138.66
non-VPDs	101.04	811.95	703.59	13.66^*^(10.04,17.68)	342.83
Respiratory route	168.3	644.77	283.11	12.75^*^(7.47,18.19)	218.14
PTB	56.08	31.58	-43.69	-2.85^*^(-4.75,-0.49)	59.26
Influenza	1.39	449.4	32230.94	31.18^*^(16.23,47.66)	48.94
Mumps	80.26	8.93	-88.87	-10.85^*^(-13.17,-8.43)	33.29
Scarlet fever	23.91	32.32	35.17	2.38(-2.98,6.8)	29.28
Rubella	1.81	0.05	-97.24	-29.56^*^(-42.68,-13.9)	15.63
Measles	4.39	0.01	-99.77	-31.41^*^(-43.94,-16.4)	3.11
Fecal-oral route	48.3	148.02	206.46	4.79^*^(0.42,9.65)	176.24
HFMD^#^	45.38	30.98	-31.73	-10.53(-20.93,1.09)	109.36
Other infectious diarrhea	1.97	98.18	4883.76	21.81^*^(13.6,29.68)	47.18
Bacillary and amebic dysentery	37.44	13.83	-63.06	-5.61^*^(-6.87,-4.22)	30.5
Hepatitis E	7.05	2.92	-58.58	-4.26^*^(-6.11,-3.09)	3.75
Hepatitis A	1.81	1.72	-4.97	-2.06(-6.39,2.48)	1.69
Blood-borne/sexual contact route	139.21	83.06	-40.33	-2.52^*^(-4.11,-0.3)	83.12
Syphilis	10.25	40.01	290.34	7.75^*^(6.68,8.75)	35.93
Hepatitis B	114.86	24.94	-78.29	-7.44^*^(-9.41,-4.54)	25.49
Hepatitis C	3.21	8.88	176.64	7.91^*^(5.02,10.68)	6.94
Gonorrhea	5.13	3.16	-38.4	-3.36^*^(-4.96,-1.8)	5.94
HIV/AIDS	0.36	5.71	1486.11	15.94^*^(14.77,17.53)	5.45
Hepatitis (untyped)	5.41	0.36	-93.35	-12.88^*^(-14.04,-11.9)	3.37
Zoonotic and vector-borne route	4.88	4.32	-11.48	-0.16(-2.39,1.67)	3.96
HB	0.31	4.09	1219.35	14.50^*^(11.15,19.29)	2.85
HFRS	4.16	0.06	-98.56	-19.66^*^(-21.56,-17.74)	0.93

*HB* Human brucellosis, *HFMD* Hand, foot and mouth disease, *HFRS* Hemorrhagic fever with renal syndrome, *NIDs* Notifiable infectious diseases, *PTB* Pulmonary tuberculosis, *VPDs* Vaccine-preventable diseases

^#^HFMD data were analyzed starting in 2008. ^*^ AAPC differs significantly from 0 at the alpha = 0.05 level. Due to high data volatility, the presence of extreme values (e.g., zero counts), or, in the case of COVID-19, a limited observation period (2020–2024), the following diseases were excluded from joinpoint trend analysis as they do not support stable model fitting and are therefore not presented in the table: neonatal tetanus, schistosomiasis, human infection with a novel influenza A virus subtype, rabies, hepatitis D, JE, echinococcosis leprosy, Mpox, AHC, dengue fever, pertussis, anthrax, meningococcal meningitis, epidemic and endemic typhus, AFP, malaria, typhoid and paratyphoid fever and COVID-19

#### Sex-specific analysis

The average annual ASIR was higher in males (565.30 per 100,000) than in females (393.35 per 100,000). Both sexes experienced significant increasing trends, with AAPCs of 5.27% (95%CI: 1.28%,8.95%) for males and 7.69% (95%CI: 3.36%,11.93%) for females. The annual ASIR in males increased by 110.73%, from 444.96 to 937.66 per 100,000, while females showed a more pronounced increase of 212.33%, from 268.92 to 839.93 per 100,000.

#### Disease category analysis

The average annual ASIRs for Category B and Category C diseases were 243.40 and 238.09 per 100,000, respectively. Category B diseases showed no significant temporal trend (AAPC: 2.41%; 95%CI: -1.45%,5.00%), whereas Category C diseases exhibited a marked upward trend (AAPC: 12.75%; 95%CI: 7.47%,18.19%). The annual ASIR of Category C diseases increased dramatically by 585.49%, from 85.72 to 587.60 per 100,000.

#### VPDs and non-VPDs analysis

VPDs and non-VPDs showed contrasting trends. The average annual ASIR was 138.66 per 100,000 for VPDs and 342.83 per 100,000 for non-VPDs. VPDs declined significantly (AAPC: -6.98%; 95%CI: -8.62%,-5.30%), with a decrease of 73.73% from 259.68 to 68.23 per 100,000. In contrast, non-VPDs increased substantially (AAPC: 13.66%; 95%CI: 10.04%,17.68%), rising by 703.59% from 101.04 to 811.95 per 100,000.

#### Transmission route analysis

The average annual ASIRs by transmission route were: respiratory route (218.14 per 100,000), fecal-oral route (176.24 per 100,000), blood-borne/sexual contact route (83.12 per 100,000), zoonotic and vector-borne route (3.96 per 100,000), and other routes (0.03 per 100,000). Diseases transmitted via respiratory and fecal-oral routes showed significant upward trends, with AAPCs of 12.75% (95%CI: 7.47%,18.19%) and 4.79% (95%CI: 0.42%,9.65%), respectively. Blood-borne/sexual contact diseases exhibited a declining trend (AAPC: -2.52%; 95%CI: -4.11%, -0.30%), while zoonotic and vector-borne diseases showed no statistically significant trend.

#### Disease-specific analysis

Among respiratory route infectious diseases, PTB exhibited the highest average annual ASIR (59.26 per 100,000), followed by influenza (48.94 per 100,000) and mumps (33.29 per 100,000). Significant declining trends were observed in the annual ASIRs of PTB, mumps, rubella, and measles, with AAPCs of -2.85% (95%CI: -4.75%,-0.49%), -10.85% (95%CI: -13.17%,-8.43%), -29.56% (95%CI: -42.68%,-13.90%), and − 31.41% (95%CI: -43.94%,-16.40%), respectively. In contrast, influenza showed a marked upward trend (AAPC: 31.18%; 95%CI: 16.23%,47.66%), while no significant temporal trend was detected for scarlet fever.

For diseases transmitted via the fecal-oral route, HFMD had the highest average annual ASIR (92.97 per 100,000), followed by other infectious diarrhea (47.18 per 100,000) and bacillary and amebic dysentery (30.50 per 100,000). Declining trends were observed for dysentery and hepatitis E, with AAPCs of -5.61% (95%CI: -6.87%,-4.22%) and − 4.26% (95%CI: -6.11%,-3.09%), respectively. Other infectious diarrhea showed a substantial increase (AAPC: 21.81%; 95%CI: 13.60%,29.68%), whereas HFMD and hepatitis A exhibited no statistically significant trends.

For diseases transmitted via blood-borne/sexual contact route, syphilis exhibited the highest average annual ASIR (35.93 per 100,000 population), followed by hepatitis B (25.49 per 100,000) and hepatitis C (6.94 per 100,000). Significant declining trends were observed in the annual ASIRs of hepatitis B, gonorrhea and hepatitis (untyped), with AAPCs of -7.44% (95%CI: -9.41%,-4.54%), -3.36% (95%CI: -4.96%,-1.80%) and − 12.88% (95%CI: -14.04%,-11.90%), respectively. In contrast, syphilis, hepatitis C and HIV/AIDS demonstrated significant increasing trends, with AAPCs of 7.75% (95%CI: 6.68%,8.75%), 7.91% (95%CI: 5.02%,10.68%) and 15.94% (95%CI: 14.77%,17.53%), respectively.

Among zoonotic and vector-borne route infectious diseases, HB exhibited the highest average annual ASIR (2.85 per 100,000), followed by HFRS (0.93 per 100,000). A pronounced declining trend of annual ASIR was observed for HFRS (AAPC: -19.66%; 95%CI: -21.56%,-17.74%), whereas HB increased significantly (AAPC: 14.50%; 95%CI: 11.15%,19.29%).

## Discussion

In this study, NNDRS data from January 1,2005 to December 31,2024 were extracted to describe the incidence and trends of NIDs in Shenyang, China. In our study, 1,733 duplicate case reports were identified, accounting for approximately 2.60‰ (1,733/668,476) of all reported cases, indicating potential over-reporting across healthcare facilities and underscoring the need for unique patient identifiers and real-time duplicate checks. Additionally, 57,483 initially reported cases were subsequently reclassified as other conditions, reflecting diagnostic discrepancies between preliminary clinical assessments and final confirmations that highlight challenges in the early recognition and differential diagnosis of infectious diseases.

The study revealed that from 2005 to 2024, NIDs in Shenyang were predominantly Category B (58.82%), followed by Category C (41.18%), whereas Category C diseases were more common nationwide during the same period [21, 22]. It was also found that in Shenyang, respiratory transmission was the primary route of infection (47.41%), in contrast to the national pattern where fecal-oral transmission predominated, accounting for 47.83% of total cases [21, 22]. These findings indicate that the epidemiological profile of NIDs in Shenyang differs from the overall national pattern, particularly in the hierarchy of transmission routes. Therefore, while adhering to the national prevention and control framework, Shenyang should implement more targeted strategies to enhance surveillance and management of locally predominant NIDs.

This study found that the spectrum of NIDs in Shenyang has undergone significant shifts over time. Between 2005 and 2019, the dominant transmission routes gradually shifted from primarily blood-borne/sexual and respiratory transmission to intestinal and respiratory transmission, a trend consistent with national findings [21, 22]. Since 2020, the distribution of transmission routes has changed anew, with a rebound in the proportion of blood-borne/sexual and respiratory diseases. After 2022, the proportion of respiratory infectious diseases increased markedly, surpassing 70% in both 2023 and 2024. This shift is closely associated with three key factors: (1) the strict COVID-19 isolation and control measures implemented in China from 2020 onward, which constrained healthcare-seeking behaviors and altered the surveillance and reporting landscape for some diseases [34, 35]; (2) the inclusion of influenza antigen-positive cases as confirmed diagnoses in the Diagnosis and Treatment Protocol for Influenza (2020 Edition), which enhanced influenza surveillance sensitivity [36]; and (3) the significant rise in reported COVID-19 cases following the discontinuation of isolation measures in late 2022.

This study also observed that the decline in the proportion of Category B diseases and the sharp decrease in VPDs in 2023 and 2024 may also be related to the three factors mentioned above. Notably, the period of compositional change in NIDs coincides with major phases of adjustment in control policies and surveillance standards, underscoring the complex interplay between the natural dynamics of diseases and public health interventions [37].

The study found that from 2005 to 2024, the average annual ASIRs of Category B and Category C infectious diseases in Shenyang were similar, at 243.40/100,000 and 238.09/100,000, respectively. The combined overall average annual ASIR was 481.49/100,000, which was lower than the national average for the same period [21, 22]. By transmission route, the average annual ASIR of respiratory infectious diseases in Shenyang was 218.14/100,000, higher than the national level. In contrast, the rates for fecal-oral transmission diseases (176.24/100,000), blood-borne/sexually transmitted diseases (83.12/100,000), as well as zoonotic and vector-borne diseases (3.96/100,000), were all lower than the corresponding national averages [21, 22]. These findings suggest that while the overall burden of NIDs in Shenyang is relatively low nationwide, the burden of respiratory infectious diseases remains substantial and warrants focused attention.

In this study, the annual ASIR of NIDs in Shenyang showed an overall increasing trend from 2005 to 2024, primarily driven by a rise in Category C diseases, consistent with national observations from the same period [21, 22]. The increase in Category C disease incidence was largely attributable to rising rates of influenza and other infectious diarrhea, which may also explain the concurrent upward trends observed for respiratory and fecal-oral transmission diseases in Shenyang.

Distinct regional patterns emerged when comparing Shenyang to national data [21, 22]. While Shenyang experienced a rising trend in the incidence of respiratory infectious diseases, no significant nationwide increase was observed [21, 22]. The incidence of fecal-oral transmission diseases in Shenyang showed an upward trend, consistent with the national pattern [21, 22]. In contrast, the incidence of blood-borne/sexually transmitted diseases exhibited a declining trend in Shenyang, whereas no clear decline was evident at the national level [21, 22]. The incidence of zoonotic and vector-borne diseases remained stable in Shenyang, mirroring the national trend [21, 22]. This overall stability may be related to the opposing trends between the two main diseases in this category: a significant increase in HB and a marked decline in HFRS, which offset each other in the aggregated data. These comparisons suggest that Shenyang has achieved relatively significant success in controlling blood-borne/sexually transmitted diseases, yet underscores the need to strengthen prevention and control measures targeting respiratory transmission.

Notably, the incidence of VPDs in Shenyang exhibited a marked decrease, largely attributable to significant declines in PTB, measles, rubella, mumps, and hepatitis B. This declining trend was consistent with the goals and timeline of the national immunization program, suggesting a possible beneficial impact of vaccination strategies, which was consistent with previous studies conducted both in China and globally [8–12, 38–40].

In this study, the top ten NIDs reported in Shenyang between 2005 and 2024 were, in descending order: PTB, HFMD, influenza, syphilis, COVID-19, other infectious diarrhea, hepatitis B, bacillary and amebic dysentery, mumps, and scarlet fever. Collectively, these diseases accounted for 88.62% of all reported cases in the city. Trend analysis revealed declining patterns for PTB, hepatitis B, bacillary and amebic dysentery, and mumps, while no clear upward trend was observed for HFMD or scarlet fever. The increase in reported influenza cases was largely attributed to a surge following updates in surveillance criteria [36]. The rise in other infectious diarrhea was concentrated between 2005 and 2007, and that of syphilis between 2005 and 2013; in each case, the annual ASIR plateaued thereafter. Although a substantial increase in COVID-19 cases was reported in 2023 and 2024, surveillance data from 2025 indicated a subsequent decline. Overall, the increase in the annual ASIR of NIDs in Shenyang is largely attributable to the increases observed during certain periods and modifications in surveillance criteria. Consequently, the overall epidemic situation of NIDs in the city remains within a manageable range.

In spite of the above findings, the limitations in our study should be considered. First, not all NIDs cases were reported to NNDRS system, analysis based on the NIDRIS data may reflect the level of reported cases in Shenyang, but may not be fully representative of local NIDs incidence. This gap stems mainly from two sources: (1) only cases presenting to healthcare facilities are reported, those not seeking medical care remain unrecorded, and (2) underreporting occurs even among cases that reach hospitals. However, the proportion of cases not seeking medical care cannot be currently assessed, due to the passive nature of the surveillance system [41]. In the future, we will conduct an active surveillance to estimate this proportion. While underreporting rate surveys in hospitals have been conducted in other Chinese provinces, the data reveal considerable variation across years and regions, with rates ranging from 1.09% to 9.81% [42–46]. We will also conduct a local survey on underreporting rate in the local hospitals in Shenyang. Second, the isolation measures implemented during the COVID-19 pandemic may have disrupted routine healthcare-seeking behavior for other infectious diseases, potentially affecting the objective assessment of their epidemiological trends. Third, only annual incidence data were used in this study to evaluate the changing trends of NIDs over the past 20 years. In the future, we will further explore the epidemiological trends at seasonal and monthly levels to better characterize transmission dynamics. Fourth, surveillance data for COVID-19 only covers the period 2020–2024, while including these data is essential for accurately representing the contemporary disease spectrum, their inclusion disproportionately influences the overall incidence trend in the latter part of the study period. Finally, this study employed ASIRs to control for demographic changes when comparing trends over time. The absence of age-stratified analyses, however, limited the ability to determine which specific age groups carried the highest disease burden, to examine whether trends varied across different age cohorts, or to evaluate the potential influence of changes in Shenyang’s population age structure. In the future, we will also conduct more detailed age-specific analyses to explore these aspects.

## Conclusions

The overall incidence of NIDs in Shenyang was lower than the national average, however, the disease burden of respiratory infections is relatively prominent and should become a key focus for future prevention and control. The observed upward trend in overall incidence may be influenced by the increases observed during certain periods and modifications in surveillance criteria, indicating that the epidemic situation remains manageable. It is noteworthy that the significant decline in the incidence of VPDs suggests a possible beneficial impact of immunization programs, and such interventions should be consolidated and strengthened.

## Data Availability

The reported cases data that support the findings of this study were downloaded from China’s NNDRS, a non-commercial national surveillance platform accessible only with authorization and not publicly available. The processed datasets necessary to reproduce the study’s findings are, however, available from the corresponding author upon reasonable request.

## Abbreviations

AAPC: Average annual percent change
AFP: Acute flaccid paralysis
AHC: Acute hemorrhagic conjunctivitis
APC: Annual percent change
ASIR: Age-standardized incidence rates
HB: Human brucellosis
HFMD: Hand, foot and mouth disease
HFRS: Hemorrhagic fever with renal syndrome
JE: Japanese encephalitis
Mpox: Monkeypox
NIDs: Notifiable infectious diseases
NNDRS: National notifiable diseases reporting system
NPIs: Non-pharmaceutical interventions
PTB: Pulmonary tuberculosis
SARS: Severe acute respiratory syndrome
VPDs: Vaccine-preventable diseases

## References

[1] Keddy KH, Gobena T. The continuing challenge of infectious diseases[J]. Lancet Infect Dis. 2024;24(8):800–1. 10.1016/S1473-3099(24)00219-6. 38640939 10.1016/S1473-3099(24)00219-6

[2] Liao C, Wang B, Lyu J, et al. Growing global public health challenges[J]. Chin J Epidemiol. 2025;46(1):1–8. 10.3760/cma.j.cn112338-20241230-00839. (In Chinese). 10.3760/cma.j.cn112338-20241230-00839 39828540

[3] Peter J. The Global Challenge of Viral Infections: A Growing Threat to Public Health[J]. Int J Collab Res Intern Med Public Health. 2024;16,(3):1–2. 10.35248/2332-2594.24.16.03.001-002.

[4] Wang S, Li W, Wang Z, et al. Emerging and reemerging infectious diseases: global trends and new strategies for their prevention and control[J]. Signal Transduct Target Ther. 2024;9(223):1–68. 10.1038/s41392-024-01917-x. 39256346 10.1038/s41392-024-01917-x PMC11412324

[5] WHO. Global Health Estimates. Life expectancy and leading causes of death and disability. https://www.who.int/data/gho/data/themes/mortality-and-global-health-estimates. Accessed on December 20, 2025.

[6] Su B, Talifu Z, Feng Z. Epidemiological Shifts in Infectious Diseases in China: Implications and Policy Recommendations[J]. China CDC Wkly. 2023;5(42):948–51. 10.46234/ccdcw2023.178. 38026098 10.46234/ccdcw2023.178 PMC10646164

[7] Liu Q, Liu M, Liang W, et al. Global distribution and health impact of infectious disease outbreaks, 1996–2023: a worldwide retrospective analysis of World Health Organization emergency event reports[J]. J Glob Health. 2025;15(04151):1–12. 10.7189/jogh.15.04151. 10.7189/jogh.15.04151 PMC12082254 40375732

[8] Wu Q, Zaid M, Xuan Z, et al. Changes in epidemiological features of vaccine preventable infectious diseases among three eras of national vaccination strategies from 1953 to 2018 in Shanghai, China[J]. Lancet Reg Health West Pac. 2021;7(100092):1–11. 10.1016/j.lanwpc.2021.100092. 10.1016/j.lanwpc.2021.100092 PMC8315356 34327419

[9] Yu W, Lee L, Liu Y, et al. Vaccine-preventable disease control in the People’s Republic of China: 1949–2016[J]. Vaccine. 2018;36(52):8131–7. 10.1016/j.vaccine.2018.10.005. 30497834 10.1016/j.vaccine.2018.10.005 PMC7115483

[10] Pezzotti P, Bellino S, Prestinaci F, et al. The impact of immunization programs on 10 vaccine preventable diseases in Italy: 1900–2015[J]. Vaccine. 2018;36(11):1435–43. 10.1016/j.vaccine.2018.01.065. 29428176 10.1016/j.vaccine.2018.01.065

[11] Jit M, Dang TT, Friberg I, et al. Thirty years of vaccination in Vietnam: impact and cost-effectiveness of the national expanded program on immunization[J]. Vaccine. 2015;33(Suppl 1):A233–9. 10.1016/j.vaccine.2014.12.017. 25919167 10.1016/j.vaccine.2014.12.017 PMC4428532

[12] Deng LL, Han YJ, Li ZW, et al. Epidemiological characteristics of seven notifiable respiratory infectious diseases in the mainland of China: an analysis of national surveillance data from 2017 to 2021[J]. Infec Dis Poverty. 2023;12(99):1–17. 10.1186/s40249-023-01147-3. 10.1186/s40249-023-01147-3 PMC10642048 37953290

[13] Li T, Qiang N, Bao Y, et al. Global burden of enteric infections related foodborne diseases, 1990–2021: findings from the Global Burden of Disease Study 2021[J]. Sci One Health. 2024;10(3):100075. 10.1016/j.soh.2024.100075. 10.1016/j.soh.2024.100075 PMC11402448 39282625

[14] Esin S, Bono LD, Pistello M. Sexually Transmitted Infections: Global Trends, Diagnostic Advances, and Emerging Challenges[J]. New Microbiol. 2025;48(2):113–36. PMID: 41123498. 41123498

[15] Fahme SA, Chehab S, Logie CH, et al. Intersecting social-ecological vulnerabilities to and lived experiences of sexually transmitted infections among Syrian refugee women in Lebanon: a qualitative study[J]. PLoS Global Public Health. 2024;4(8):e0003507. 10.1371/journal.pgph.0003507. 39116144 10.1371/journal.pgph.0003507 PMC11309427

[16] Unemo M, Ross J, Serwin A, et al. 2020 European guideline for the diagnosis and treatment of gonorrhea in adults[J]. Int J STD AIDS. 2020;0(0):1–17. 10.1177/0956462420949126. 10.1177/0956462420949126 33121366

[17] Wang CC, Zhang WX, He Y, et al. Global Epidemiology of Vector-Borne Parasitic Diseases: Burden, Trends, Disparities, and Forecasts (1990–2036)[J]. Pathogens. 2025;14(9):844. 10.3390/pathogens14090844. 41011745 10.3390/pathogens14090844 PMC12472468

[18] Riccio MD, Caini S, Bonaccorsi G, et al. Global analysis of respiratory viral circulation and timing of epidemics in the pre-COVID-19 and COVID-19 pandemic eras, based on data from the Global Influenza Surveillance and Response System (GISRS)[J]. Int J Infect Dis. 2024;144:107052. 10.1016/j.ijid.2024.107052. 38636684 10.1016/j.ijid.2024.107052

[19] Liu Y, Morgenstern C, Kelly J, et al. The impact of non-pharmaceutical interventions on SARS-CoV-2 transmission across 130 countries and territories[J]. BMC Med. 2021;19(1):40. 10.1186/s12916-020-01872-8. 33541353 10.1186/s12916-020-01872-8 PMC7861967

[20] Zhang L, Wilson DP. Trends in Notifiable Infectious Diseases in China: Implications for Surveillance and Population Health Policy[J]. PLoS ONE. 2012;7(2):e31076. 10.1371/journal.pone.0031076. 22359565 10.1371/journal.pone.0031076 PMC3281048

[21] Chen HP, Chen LF, Lu SX, et al. Analysis of the incidence of notifiable infectious diseases in China from 2005 to 2020[J]. Chin J Urban Rural Enterp Hygiene. 2021;8(8):111–5. 10.16286/j.1003-5052.2021.08.042 . (In Chinese).

[22] He WM, Yu XB, Wang HT, et al. Analysis of the incidence and characteristics of legal infectious diseases in China from 2014 to 2024[J]. Infect Dis Info. 2025;38(5):484–90. 10.3969/j.issn.1007-8134.2025.05.012 . (In Chinese).

[23] Zhou QY, Ma T, Zhao YY, et al. Trends in incidence of notifiable infectious diseases in Nanjing City from 2004 to 2022[J]. China Prev Med J. 2025;37(5):476–80. 10.19485/j.cnki.issn2096-5087.2025.05.009 . (In Chinese).

[24] Ma X, Liu XY, Fu YQ, et al. Analysis on the epidemic trend of notifiable infectious diseases in Henan Province from 2004 to 2022[J]. Mod Prev Med. 2024;51(3):529–35. 10.20043/j.cnki.MPM.202311218 . (In Chinese).

[25] Lessania MN, Li Z, Jing F, et al. Human mobility and the infectious disease transmission: a systematic review[J]. Geo-Spat Inf Sci. 2024;27(6):1824–51. 10.1080/10095020.2023.2275619. 40046953 10.1080/10095020.2023.2275619 PMC11882145

[26] Zhou HR, Wang XL, Li GF, et al. The epidemiological trends of 40 national notifiable infectious diseases in China: An analysis of national surveillance data from 2013 to 2022. One Health Bull. 2025;5(1):21–32. 10.4103/ohbl.ohbl-26-24.

[27] Dai MY. Analysis on epidemiological characteristics of notifiable infectious diseases, Shenyang city,2013–2017 [J]. Prev Med Trib. 2019;25(2):104–8. 10.16406/j.pmt.issn.1672-9153.2019.02.010 . (In Chinese).

[28] National Disease Control and Prevention Administration. Notifiable Infectious Diseases List. https://www.ndcpa.gov.cn/jbkzzx/c100041/common/list.html. Accessed on December 20, 2025. (In Chinese).

[29] Wang CC, Prather KA, Josué S, et al. Airborne transmission of respiratory viruses[J]. Science. 2021;373(6558):eabd9149. 10.1126/science.abd9149. 34446582 10.1126/science.abd9149 PMC8721651

[30] Stürchler D. Infections transmitted via the fecal-oral route: a simple score for a global risk map[J]. J Travel Med. 2023;30(6):taad069. 10.1093/jtm/taad069. 37158467 10.1093/jtm/taad069 PMC10628772

[31] Shiferaw W, Martin BM, Dean JA, et al. A systematic review and meta-analysis of sexually transmitted infections and blood-borne viruses in travelers[J]. J Travel Med. 2024;31(4):taae038. 10.1093/jtm/taae038. 38438164 10.1093/jtm/taae038 PMC11149723

[32] Kulkarni MA, Berrang-Ford Lea, Buck PA, et al. Major emerging vector-borne zoonotic diseases of public health importance in Canada[J]. Emerg Microbes Infect. 2015;4(6):e33. 10.1038/emi.2015.33. 26954882 10.1038/emi.2015.33 PMC4773043

[33] Morchón R, Bueno-Marí R, Bravo-Barriga D, Biology. Control and Zoonotic Role of Disease Vectors[J]. Pathogens. 2023;12(6):797. 10.3390/pathogens12060797. 37375487 10.3390/pathogens12060797 PMC10304399

[34] Li J, Yuan F, Fan S, et al. The impact of COVID-19 pandemic on reported notifiable infectious diseases in China: An interrupted time series analysis[J]. Am J Infect Control. 2025;53(3):340–7. 10.1016/j.ajic.2024.10.010. 39454757 10.1016/j.ajic.2024.10.010

[35] Zhang Y, Feng W. Impact of the coronavirus disease 2019 pandemic on the diversity of notifiable infectious diseases: a case study in Shanghai, China[J]. Peer J. 2024;12(12):e17124. 10.7717/peerj.17124. 38495754 10.7717/peerj.17124 PMC10941765

[36] National Health Commission of the People’s Republic of China. Circular on Issuing the Diagnosis and Treatment Protocol for Influenza. 2020 Edition. https://www.nhc.gov.cn/ylyjs/zcwj/202011/472e0ad873014c168dbe22e19d605e20.shtml. Accessed on December 20, 2025. (In Chinese).

[37] Jiang Y, Dou XF, Yan CQ, et al. Epidemiological characteristics and trends of notifiable infectious diseases in China from 1986 to 2016. J Glob Health. 2020;10(2):020803. 10.7189/jogh.10.020803. 33214900 10.7189/jogh.10.020803 PMC7649044

[38] Li ZW, Yin CN, Wang HT, et al. Incidence trend and disease burden of seven vaccine-preventable diseases in Shandong province, China, 2013–2017: Findings from a population-based observational study[J]. Vaccine X. 2022;5(10):100145. 10.1016/j.jvacx.2022.100145. 10.1016/j.jvacx.2022.100145 PMC8867126 35243321

[39] Song Q, Li Y, Cao L, et al. Impact of National Immunization Strategies on Vaccine-Preventable Diseases-China, 1950–2021[J]. China CDC Wkly. 2024;6(16):339–43. 10.46234/ccdcw2024.064. 38736466 10.46234/ccdcw2024.064 PMC11082052

[40] Pan J, Wang Y, Cao L, et al. Impact of immunization programs on 11 childhood vaccine-preventable diseases in China: 1950–2018[J]. Innovation. 2021;2(2):100113. 10.1016/j.xinn.2021.100113. 34557762 10.1016/j.xinn.2021.100113 PMC8454656

[41] Li X, Chang HH, Cheng Q, et al. A spatial hierarchical model for integrating and bias-correcting data from passive and active disease surveillance systems[J]. Spat Spatiotemporal Epidemiol. 2020;35:100341. 10.1016/j.sste.2020.100341. 10.1016/j.sste.2020.100341 PMC7704115 33138957

[42] Wang M, Xin ZJ, Xie JQ, et al. Investigation and analysis on the quality and omission report of statutory infectious diseases in medical institutions in Fengtai District of Beijing from 2014 to 2017[J]. J Med Pest Control. 2019;35(12):1131–4. 10.7629/yxdwfz201912003 . (In Chinese).

[43] Li YS, Ma BZ, Shi Y, et al. The investigation and analysis of missed reporting of notifiable diseases in medical institutions in Qinghai Province between 2009 and 2011[J]. Mod Prev Med. 2014;41(2):340–2. doi:CNKI:SUN:XDYF.0.2014-02-049. (In Chinese).

[44] Huo F, Xu J, Xia WD, et al. Underreporting of notifiable communicable diseases in medical institutions in Tianjin,2004–2012[J]. Dis Surveill. 2013;28(11):943–6. 10.3784/j.issn.1003. (In Chinese).

[45] Ma LZ, Luo XS, Yang CH, et al. Assessment on reporting rates of notifiable infectious disease in medical institutions in Sichuan province in 2015[J]. J Prev Med Inf. 2017;33(3):247–51. doi:CNKI:SUN:YFYX.0.2017-03-013. (In Chinese).

[46] Liu ZT, Li QF, Wang RH, et al. Underreporting of notifiable communicable diseases in medical institutions in Yunnan province,2010[J]. Dis Surveill. 2011;26(7):565–7. 10.3784/j.issn.1003-9961.2011.07.019 . (In Chinese).

